# Primary cilia elongation in response to interleukin-1 mediates the inflammatory response

**DOI:** 10.1007/s00018-012-0980-y

**Published:** 2012-04-06

**Authors:** A. K. T. Wann, M. M. Knight

**Affiliations:** Biomedical Engineering, 2nd Floor Cell and Tissue Laboratories, School of Engineering and Materials Science, Queen Mary, University of London, Mile End Road, London, E1 4NS UK

**Keywords:** Primary cilia, Inflammation, Cytokine, Protein kinase A, Chondrocyte

## Abstract

Primary cilia are singular, cytoskeletal organelles present in the majority of mammalian cell types where they function as coordinating centres for mechanotransduction, Wnt and hedgehog signalling. The length of the primary cilium is proposed to modulate cilia function, governed in part by the activity of intraflagellar transport (IFT). In articular cartilage, primary cilia length is increased and hedgehog signaling activated in osteoarthritis (OA). Here, we examine primary cilia length with exposure to the quintessential inflammatory cytokine interleukin-1 (IL-1), which is up-regulated in OA. We then test the hypothesis that the cilium is involved in mediating the downstream inflammatory response. Primary chondrocytes treated with IL-1 exhibited a 50 % increase in cilia length after 3 h exposure. IL-1-induced cilia elongation was also observed in human fibroblasts. In chondrocytes, this elongation occurred via a protein kinase A (PKA)-dependent mechanism. G-protein coupled adenylate cyclase also regulated the length of chondrocyte primary cilia but not downstream of IL-1. Chondrocytes treated with IL-1 exhibit a characteristic increase in the release of the inflammatory chemokines, nitric oxide and prostaglandin E2. However, in cells with a mutation in IFT88 whereby the cilia structure is lost, this response to IL-1 was significantly attenuated and, in the case of nitric oxide, completely abolished. Inhibition of IL-1-induced cilia elongation by PKA inhibition also attenuated the chemokine response. These results suggest that cilia assembly regulates the response to inflammatory cytokines. Therefore, the cilia proteome may provide a novel therapeutic target for the treatment of inflammatory pathologies, including OA.

## Introduction

Primary cilia are single finger-like projections that extend several microns into the extracellular environment and are expressed by the majority of eukaryotic cells. Despite their discovery more than a century ago, only in the last few decades has evidence been gathered to highlight the role that this tubulin-based structure plays in many facets of cell biology, including differentiation and vertebrate development [1, 2], cell cycle control [3], cancer signaling [4], sensory function and migration [5], and mechanotransduction [6–9]. The primary cilia axonome is extended from the basal body upon entry to G0, and its genesis, maintenance, and function rely upon intraflagellar transport (IFT) [10]. The large number of ciliopathies have served both to emphasize the physiological importance of the primary cilium and to provide models to better understand their function [11]. Many of these pathologies are associated with alterations in cilia length [12], adding support to the concept of a cilia structure–function relationship [13–15].

Axonomal length control has been extensively studied in model systems such as *Chlamydomonas* [16–18] and *C*. *elegans* [17, 19] since 1969. Mechanisms acting to regulate flagellar length include Ca^2+^ concentration [20] and protein phosphorylation [21]. A correlation between cilia length and IFT particle size has also been observed [18]. Study of ciliary length in mammalian cells has indicated many factors regulating axonomal length. These include reduced intracellular calcium and increased cyclic AMP (cAMP) acting to elongate cilia, through a PKA-dependent increase in anterograde (towards the tip) IFT [14]. Molecular approaches have identified the involvement in cilia length control of molecules engaged in the organization of the actin cytoskeleton and in soluble tubulin levels [22, 23]. Most recently, work in vascular endothelium has elucidated roles for protein kinase C (PKC) and mitogen-activated (MAP) protein kinases [15]. Cilia elongation receives ever-increasing interest, as reviewed recently [24].

A large contingent of diseases including arthritis, arthrosclerosis, and cancer involve inflammation. In articular cartilage, where primary cilia protrude into the extracellular matrix [25], the degenerative condition of OA is associated with increases in cilia length and prevalence [26], and the increased expression of hedgehog signaling genes [27]. More broadly, studies in kidney epithelium suggest that cilia length changes post-injury are important in the process of renal repair [28]. Cytokines are involved in a huge range of physiological and pathological processes [29]. In inflammatory pathologies, the quintessentially pro-inflammatory cytokine Interleukin-1β (IL-1β) and its receptors are up-regulated as part of the broad spectrum of inflammatory mediators activated in many cell types. As such, we hypothesized that IL-1 exposure increases cilia length and that the cilium is involved in inflammatory signaling. We show that primary cilia length is increased by IL-1 and that cilia elongation drives the downstream inflammatory response in the form of chemokine release. This suggests, for the first time, that primary cilia and IFT play an important role in inflammation. These studies, therefore, open the door to a host of new therapeutic targets for a wide variety of inflammatory pathologies.

## Materials and methods

### Cell culture

Bovine forefeet from 18-month-old steers were obtained fresh from slaughter from a local abattoir and primary chondrocytes isolated by enzymatic digestion as previously described [30]. Cells were cultured in Dulbecco’s Modified Eagles Medium (DMEM; Sigma-Aldrich, Poole, UK) supplemented with 10 % (v/v) fetal calf serum (FCS), 1.6 mM l-glutamine, 81 µ mL^−1^ penicillin, 80 μg mL^−1^ streptomycin, 16 mM HEPES buffer, and 0.68 mM 1-ascorbic acid (all Sigma-Aldrich). Cells were seeded onto FCS-coated glass coverslips at 6 × 10^4^ cells cm^2^ and cultured for 5 days to attach.

Tg737^*ORPK*^ (heterozygous) mutant mice lines were generated as previously described [31]. Mice were maintained on a mixed genetic background according to approved protocols at the Medical University of South Carolina. Heterozygous *ORPK* mice were bred with heterozygous Immortomouse mice (*H*-*2* *Kb*-tsA58) which harbor a temperature sensitive SV40 large T antigen transgene under the control of an interferon-γ-inducible *H*-*2* *Kb* promoter (*H*-*2* *Kb*-tsA58) to produce *orpk*/Immortomouse compound heterozygous mice [32]. Heterozygous *ORPK* females were bred with heterozygous/Immortomouse *orpk* males. Chondrocytes were isolated from the sternum of 4-day-old mice by digestion with collagenase type II (2 mg mL^−1^) dispersed in DMEM at 37 °C for 4 h. All mice were genotyped by PCR from tail biopsy DNA. Western blot analysis was conducted to confirm the expression of SV40 large T antigen protein in chondrocytes in the presence of IFN-γ at 33 °C. Cells were cultured in DMEM supplemented with 10 % FCS, 88 U mL^−1^ penicillin, 90 μg mL^−1^ streptomycin, 10 ng mL^−1^ INF-γ, and 2.5 mM l-glutamine. Immortalized cells were grown to 90 % confluence in 5 % CO_2_/33 °C plus 10 nM IFN-γ, then cultured in non-permissive conditions at 37 °C (-IFN-γ) for 4 days before seeding onto glass FCS-coated coverslips at 6 × 10^4^ cells cm^2^. Proliferation rates (as assessed by ki-67 staining) were very low during the 48-h treatment period. This was associated with no statistically significant variation in cell number between treatments as indicated by a fluorescence DNA quantification assay using Hoescht 33258.

NIH3T3 cells were cultured in DMEM supplemented with 10 % FCS, 88 U mL^−1^ penicillin, 90 μg mL^−1^ streptomycin, and 2.5 mM l-glutamine. Cultures were serum starved for 12 h to encourage cilia expression and near-abolish proliferation prior to exposure to IL-1.

### Interleukin treatment

IL-1β was obtained from Peprotech (London, UK), and reconstituted to 1 mg mL^−1^ from solid lyophilized sterile powder in distilled water. This stock was then added to serum-supplemented DMEM and frozen in aliquots at 10 μg mL^−1^. When required, aliquots of IL-1β were thawed and diluted to 10 ng ml^−1^ in media.

### Immunofluorescent staining

Following fixation in 3.7 % paraformaldehyde at 37 °C for 8 min, coverslip cultures were permeabilized in 0.5 % triton and blocked with 5 % goat serum. Coverslips were incubated overnight at 4 °C with anti-acetylated α-tubulin primary antibody (clone 6-11B-1, 1:2,000; Sigma-Aldrich), washed, and incubated for 2 h at 25 °C with Alexa 488 anti-mouse conjugate (Invitrogen, UK) before mounting with a DAPI counterstain (Invitrogen). Ki-67 staining was conducted in identical fashion using mouse anti-ki-67 (Sigma-Aldrich) and an Alexa 594 anti-mouse conjugate (Invitrogen).

### Imaging and cilia length measurements

**Fig. 1:**
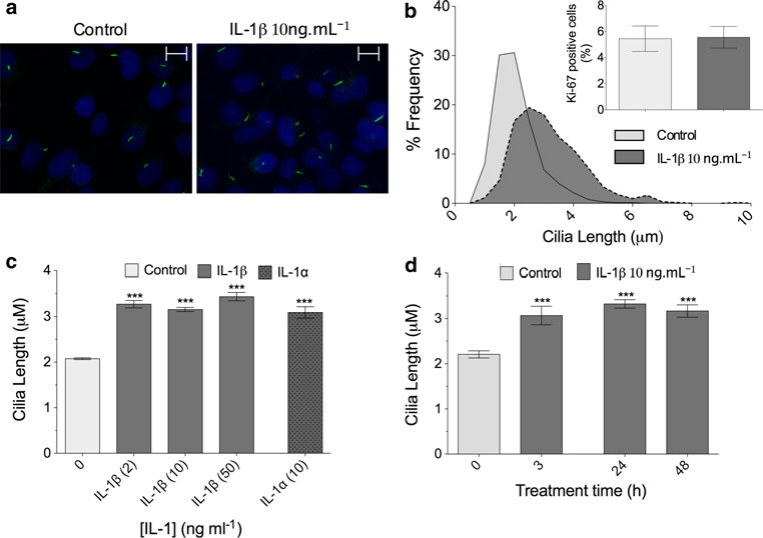
IL-1 increases primary cilia length in chondrocytes. **a** Chondrocyte primary cilia (*green*, anti-acetylated-α-tubulin, *blue* DAPI nuclei). *Scale bar* 10 μm. **b** Frequency histogram of chondrocyte cilia length showing elongation of cilia associated with IL-1β treatment (10 ng mL^−1^, 24 h). *Insert* shows that negligible cells were in a proliferative state as indicated by nuclear ki-67 expression. **c** IL-1β at 2–50 ng mL^−1^ induced statistically significant increases in cilia length as did IL-1α at 10 ng mL^−1^, but no statistically significant differences were seen between the different concentration or type of IL-1 used. **d** The influence of IL-1 was observed at 3 h and the effect maintained throughout a 48-h treatment period. Statistically significant differences are indicated relative to untreated control

A Leica SP2 confocal microscope was used to create maximum projections of confocal *z*-stacks (Fig. 1a) from which cilia length was measured using image J software. At least three different mounted preparations were used to capture five fields of cells at ×63 magnification giving data for >100 cilia per subgroup. Confocal *z* maximum projections were also used to assess cilia prevalence and ki-67 nuclear staining.

### Pharmacological agonists/antagonists

All experiments to investigate mechanism were conducted over 24 h with 10 ng mL^−1^ IL-1β. All reagents from Merck Chemicals, UK. Table 1 shows the concentrations and mode of action of the various agonists and antagonists used throughout this study.

**Table 1:** The concentrations, modes of action and summary effects of pharmacological agonists/antagonists

Drug name	Abbreviation	Concentration	Mode of action	Effect on cilia length	Effect on IL-1 elongation
PKI (14–22 amide inhibitor)	PKI	10 μM	Peptide PKA inhibitor	No effect	Complete inhibition
H89	H89	0.5 μM	ATP-site inhibitor of PKA	No effect	Complete inhibition
Bisindolylmaleimide I	BIM1	0.5 μM	ATP-site inhibitor of PKC	No effect	Partial inhibition
PD98059	PD98059	10 μM	MEK–ERK inhibitor	No effect	Complete inhibition
8-Bromo-adenosine	8-Br	10–100 μM	cAMP analogue-Activates PKA	Increase	Supplementary
2′-5′ dideoxyadenosine^a^	DOA	10 μM	p-site inhibitor of adenylate cyclase	Increase	Supplementary
Pertussis toxin^b^	PT	1 μg mL^−1^	ADP-Ribosylation of Gαi subunit	Increase	No net effect

^a^Dideoxyadenosine was additionally re-dosed after 12 h. Vehicles at equal volume were added to untreated controls. A summary of effects both on baseline and IL-1-treated ciliary length has been included

^b^Pertussis toxin was added to cultures 6 h before IL-1 treatment. All other agents were added at the same time-points and for 24 h

### PGE_2_ and NO quantification

A commercially available quantitative immunoassay (R&D Systems, UK) was used to quantify media PGE_2_ concentrations immediately following 48-h IL-1β treatment. Samples were diluted threefold before addition to a goat-anti-mouse microplate. Addition of a horseradish peroxidase-labeled PGE_2_ ensured PGE_2_ in the sample competed for binding with a monoclonal antibody, which was quantified by measuring absorbance at 450 nm following washing and addition of a substrate solution. Results were corrected for non-specific binding and read from a PGE_2_ standard curve fitted in GraphPad Prism 5. Nitrite, the stable product of NO degradation, content was quantified in the media without dilution, after an identical 48-h experiment, by spectrometric Greiss reagent assay [33], measuring absorbance at 544 nm. The same methodologies were used for murine cells investigating the role of IFT88 and for bovine cells investigating the role of PKA.

### Statistics

Data are presented using mean ± SEM. Due to the skewed nature of the data for cilia length, non-parametric statistical analysis was used in the form of Mann–Whitney *U* tests. Unpaired Student’s *t* tests were used for analysis of nitrite and PGE_2_ release. For all statistics, a two-tailed approach was used with *p* < 0.05 indicating a statistically significant difference. In the figures, ^*^ *p* < 0.05, ^**^ *p* < 0.01, ^***^ *p* < 0.0001.

## Results

### IL-1 exposure increases primary cilia length

Freshly isolated chondrocytes expressed primary cilia with a mean length of ~2 μm in monolayer cultures, with >75 % of cilia between 1 and 3 μm long. Exposure for 24 h to IL-1β, at the commonly used experimental concentration of 10 ng mL^−1^, statistically significantly increased the length of cilia in primary chondrocytes (*p* < 0.0001, *n* = 9 independent experiments; >600 cilia; Fig. 1a). Most strikingly, IL-1 increased the number of cells with cilia >3 μm long (Fig. 1b). To assess the cell cycle status of the cells, the proliferative marker ki-67 was utilized to label cells outside of G0 phase. This indicated that only a very small proportion (<6 %) of the freshly isolated cells, were outside of quiescence and, as such, effects on cilia length were not due to alterations in proliferation (Fig. 1b, insert). The approximate 50 % increase in cilia length was also observed using IL1-β at both a lower, 2 ng mL^−1^, and a higher, 50 ng mL^−1^, concentration and also when IL-1α was used at 10 ng mL^−1^ (Fig. 1c). A similar statistically significant 50 % elongation was found after 3, 24, and 48 h exposure to IL-1β (Fig. 1d). No statistically significant differences were seen between concentrations, between the two IL-1 types or between time-points. For all comparisons, *p* < 0.0001, *n* = 3 independent experiments; >100 cilia. Cilia prevalence varied between preparations (40–60 %); however, no statistically significant differences were observed between treatment groups.

### IL-1 induction of cilia elongation also occurs in fibroblasts

**Fig. 2:**
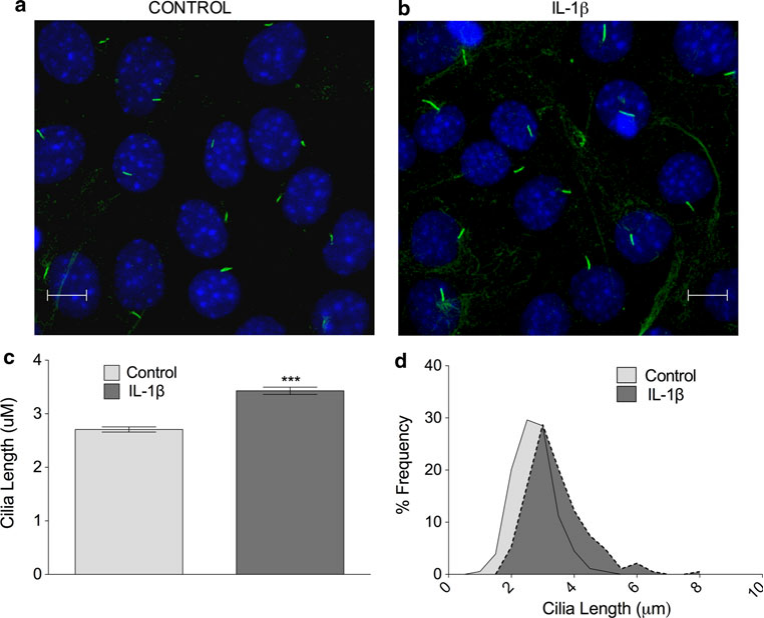
IL-1 increases primary cilia length in human fibroblasts. **a**, **b** Immunofluorescent staining of cilia in human fibroblasts (*green*, anti-acetylated-α-tubulin, *blue* DAPI nuclei). *Scale bar* 10 μm. **b**, **c** IL-1β (10 ng mL^−1^, 24 h) induced a statistically significant increase in primary cilia length in human fibroblasts. **d** Frequency histogram of fibroblast cilia length

Most eukaryotic cells express primary cilia. The cilium conducts a wide variety of roles in different cell types including epithelia [7], bone [6, 34], endothelium [15], and fibroblasts [5]. A large amount of primary cilia experiments have been conducted in the fibroblast cell line NIH3T3 [35]. In order to check if the IL-1 phenomenon was exhibited by cells other than bovine chondrocytes, we investigated IL-1 stimulation in this human fibroblast cell line. With serum-starvation and high seeding density, the cells exhibited a highly quiescent profile with 80 % cilia expression (Fig. 2a). In response to 10 ng mL IL-1, mean cilia length in NIH3T3 fibroblasts was increased by 27 % from a mean value of 2.7–3.4, the difference being statistically significant after 24 h exposure (*p* < 0.0001, *n* = 3 independent experiments; >170 cilia; Fig. 2b). This represented a more subtle, but nevertheless convincing, shift towards longer cilia (Fig. 2c) than observed in primary chondrocytes (Fig. 1b).

### IL-1 induced cilia elongation occurs via protein kinases including PKA

**Fig. 3:**
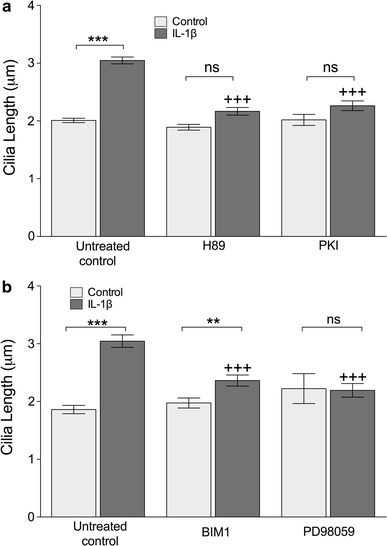
IL-1 induced cilia elongation occurs via kinase activity including PKA **a** IL-1 stimulated elongation was abolished (+++*p* < 0.0001 with respect to IL-1 only) by treatment with PKA antagonists H89 and PKI. **b** Inhibition of PKC by bisindolylmaleimide I (BIM1) resulted in the substantial inhibition of IL-1 induced elongation (+++*p* < 0.0001 with respect to IL-1 only). MEK–ERK inhibition by PD98059 abolished IL-1-induced elongation. Neither drug had an effect on cilia length in untreated cells

Previous studies in other cell types have shown the influence of adenylate cyclase, cAMP, cAMP-activated protein kinase A (PKA), and PKC and MAP kinases on primary cilia length [14, 15]. First, a role for PKA in IL-1 stimulated elongation was tested using PKA inhibitors PKI and H89 (Fig. 3a). Both drugs prevented cilia elongation associated with 24 h exposure to IL-1β (10 ng.mL^−1^) such that increases in length with IL-1β treatment were not statistically significant (*n* = 3 independent experiments; each >100 cilia). Neither inhibitor elicited an effect on cilia length when used without IL-1 (Fig. 3a).

Second, using the same pharmacological inhibitors as previous studies investigating PKC and MEK–ERK, bisindolylmaleimide I or BIM1 (0.5 μM) and PD98059 (10 μM), respectively, we investigated the importance of these kinases in IL-1β-stimulated cilia elongation. Whilst neither inhibitor influenced control of cilia length without IL-1, both statistically significantly reduced (*p* < 0.0001) the response to IL-1 (Fig. 3b). BIM1 had a partially inhibitory effect, but in the case of PD98059, the IL-1β-induced cilia elongation was completely abolished. For all experiments, *n* = 3 independent experiments; >100 cilia.

### cAMP level also regulates chondrocyte cilia length via PKA but not in response to IL-1

**Fig. 4:**
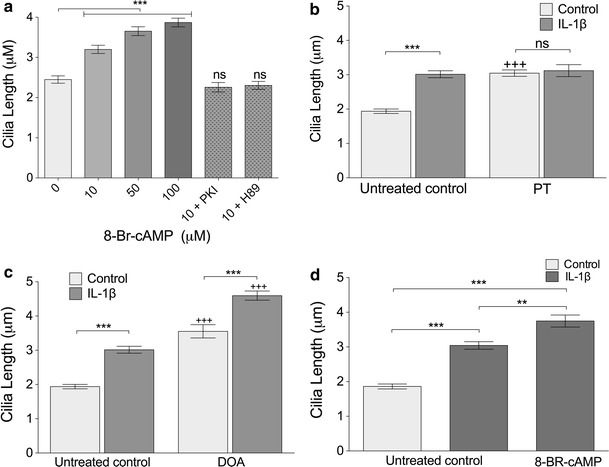
The influence of Gαi, adenylate cyclase and cAMP on cilia length appears distinct from that of IL-1. **a** 8-BR-cAMP treatment increased primary cilia length compared with untreated controls. The effect of 8-BR-cAMP was abolished by PKA antagonists PKI and H89 such that there were no significant differences with the untreated controls. **b** G-protein inhibition with pertussis toxin (PT) increased cilia length in the absence of IL-1β (+++*p* < 0.0001). There was no significant difference between IL-1β- and PT-treated and IL-1 alone. **c** Adenylate cyclase inhibitor 2′-5′deoxyadenosine (DOA) increased cilia length in both control and IL-1-treated samples relative to corresponding controls (+++*p* < 0.0001). **d** IL-1 and 8-BR-cAMP combined further increased cilia length beyond that of IL-1β alone

In light of the PKA results, we next investigated the effect of the cAMP analogue 8-Br-cAMP on cilia length in chondrocytes. Addition of this cAMP analogue at 10 μM produced a statistically significant increase in cilia length after 24 h treatment (31 %, *p* < 0.0001; Fig. 4a). 8-Br-cAMP elicited this effect with increasing potency from 10 to 100 μM (*p* < 0.0001, Kruskal–Wallis test, *n* = 3 independent experiments; >100 cilia). With 100 μM treatment, cilia length increased within 3 h (a 39 % increase in mean, *p* < 0.0001), but most striking results were seen at 24 h (a 75 % increase in mean, *p* < 0.0001, time-course data not shown). The effect of 10 μM 8-BR-cAMP was abolished by addition of either the PKA inhibitor 14–22 amide fragment, PKI (10 μM) or PKA inhibitor H89 (0.5 μM) such that cilia length was not statistically significantly from control (Fig. 4a). As such, cAMP-PKA regulation of cilia length is shown to be active in chondrocytes.

Given the ability of cAMP to elicit cilia lengthening, we next hypothesized that IL-1 may be eliciting its influence via G protein activation of adenylate cyclase and resulting conversion of ATP to cAMP. To investigate the roles of G proteins and adenylate cyclase (AC), cultures were treated with the G-protein inhibitor, pertussis toxin (PT) and the p-site adenylate cyclase antagonist, 2′-5′deoxyadenosine (DOA). Pertussis toxin had a stimulatory influence on cilia length with a 57 % increase in mean length (*p* < 0.0001). No additional effect was seen when IL-1 was added in conjunction with pertussis toxin (Fig. 4b). Inhibition of adenylate cyclase, without IL-1β, increased mean cilia length by 83 % (*p* < 0.0001). However, the effects of IL-1β and DOA were additive, such that, when cells were exposed to both, there was a 137 % increase in mean cilia length compared to the untreated control without IL-1 β (*p* < 0.0001; Fig. 4c). Thus, DOA and PT had no negative effect on the IL-1β-induced increase in cilia length which remained statistically significant (*p* < 0.0001). An inhibition of adenylate cyclase has previously been proposed as a mechanism of ciliary elongation [35], therefore, to establish if cAMP may be able to recover IL-1-induced changes, cells were treated with both IL-1 and 8-BR-cAMP (Fig. 4d). This produced a statistically significant increase in cilia length compared to IL-1β alone (*p* < 0.01). For all experiments, *n* = 3 independent experiments; >100 cilia. There is, therefore, no evidence for any link between AC-cAMP signaling and IL-1-induced cilia elongation.

### IFT88 is required for inflammatory responses to interleukin-1

**Fig. 5:**
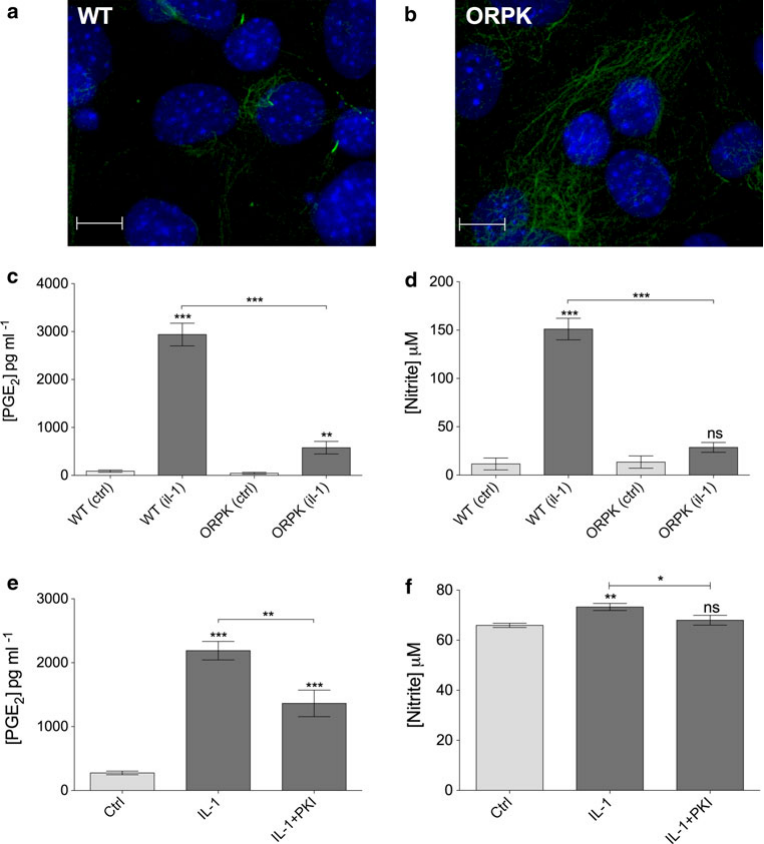
IFT88 mutation and PKA inhibition both reduce inflammatory chemokine response to IL-1. Murine WT primary cilia (**a**) (*green*, anti-acetylated-α-tubulin, *blue* DAPI nuclei). No cilia were observed in *ORPK* cells (**b**). *Scale bars* 10 μm. **c** 48 h IL-1β exposure increased PGE_2_ markedly in WT cells but to a lesser extent in ORPK cells. **d** IL-1β treatment produced a significant increase in nitrite release in WT cells but had no effect on ORPK cells. **e**, **f** IL-1β treatment produced significant increases in PGE_2_ and nitrite in bovine chondrocytes, both of which were inhibited by treatment with PKA inhibitor PKI. Data shown are mean ± SEM, *n* = 7 preparations

To assess the downstream functionality of cilia elongation in an inflammatory context, we employed a genetic approach to knockout IFT88 whilst assessing IL-1 stimulated PGE_2_ and nitrite release (indicative of nitric oxide release), classic inflammatory results of cytokine exposure in many cell types. When cultured in identical conditions to bovine primary chondrocytes wild-type (WT) murine cells exhibited approximately 40 % ciliation (Fig. 5a), whilst mutated (*ORPK)* cells exhibited no cilia (Fig. 5b). IL-1β treatment (48 h) elicited the expected large (32.9-fold) and statistically significant (*p* > 0.0001, unpaired Student’s *t* test, *n* = 7 preparations) increase in PGE_2_ in the culture medium (Fig. 5c). However, when *ORPK* cells were exposed to an identical IL-1 β treatment, the increase in PGE_2_ was muted by comparison, only a 13-fold increase, albeit still statistically significant (*p* < 0.01). The concentrations of PGE_2_ in IL-1β-treated culture samples were statistically significantly different for WT and ORPK (*p* < 0.0001). In a similar fashion, IL-1β treatment elicited a large (12.1-fold) and statistically significant (*p* < 0.0001, unpaired Student’s *t* test, *n* = 7 preparations) increase in nitrite concentration in the media. By contrast, this response was completely lost in ORPK cells where there was no statistically significant difference in nitrite concentration with and without IL-1β (Fig. 5d). Identical experiments using bovine primary chondrocytes indicate that inhibition of PKA by PKI, at a concentration that inhibited IL-1-induced cilia elongation, also statistically significantly attenuated IL-1-induced elevations in PGE_2_ and nitrite (Fig. 5e, f).

## Discussion

In this study, we present the effect of IL-1 on primary cilia length and highlight, for the first time, the potentially fundamental role of IFT-mediated primary cilia elongation in the progression of inflammation. Further to this, we show that some of the molecular mechanisms highlighted in previous studies apply to this cytokine-induced lengthening. Most interestingly of all, we demonstrate the key role for the cilium and IFT in downstream inflammatory signaling.

By their quiescent nature, freshly isolated primary chondrocytes represent an excellent and highly relevant non-proliferative primary cell model for studying ciliogenesis in the context of inflammation. Freshly isolated chondrocytes expressed primary cilia with a mean length of ~2 μm in monolayer cultures, (Fig. 1a). For comparison, in healthy bovine patellae cartilage, mean lengths of 1.1–1.5 μM, are observed in the superficial to deep zones, respectively [26]. In situ measurements of cilia length within cartilage tissue are difficult given the three dimensional orientation of the cilia and the resolution of confocal microscopy in the *z* axis. By contrast, the use of isolated chondrocytes cultured in monolayer provides a simple, accurate, and reproducible measurement of primary cilia length.

Our results indicate that the pro-inflammatory cytokine, interleukin-1, stimulates cilia elongation. It is most likely that IL-1 is having an effect on pre-existing cilia rather than holding cells in G0 for longer. Cilia length has been shown to be regulated by a host of genes, proteins, and signaling cascades [13–15, 22, 23, 35–40], but never by exposure to inflammatory cytokine. Cilia elongation occurred after just 3 h of IL-1 exposure and at the lowest concentration tested (2 ng.mL^−1^). Both IL-1β and the cell-associated form IL-1α stimulate elongation. In other tissues, fibroblasts are thought to be intermediaries in the immune reaction during an inflammatory progression that includes chemokine signaling via prostaglandins and nitric oxide release [41]. We show that this phenomenon of cilia elongation, in response to the inflammatory cytokine IL-1, is not just active in chondrocytes but is also present in fibroblasts and therefore may have implications for all inflamed tissues.

**Fig. 6:**
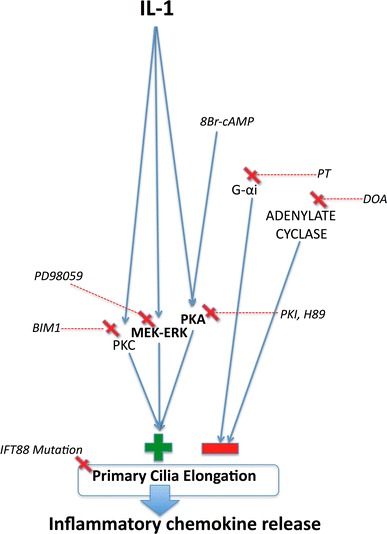
Schematic summary of the proposed pathways behind IL-1 influence on chemokine release via ciliary elongation, as indicated by pharmacological and genetic experiments. IL-1 exerts positive influence on cilia elongation via PKA, MEK–ERK, and PKC. cAMP also exerts positive influence through PKA. The adenylate cyclase system exerts negative influence by separate means. There is no evidence that cAMP or adenylate cyclase are involved in IL-1-induced ciliary elongation. IL-1-stimulated ciliary elongation via PKA is important to inflammatory signaling

Previous pharmacological work in multiple cell types [14, 15, 35] has indicated a role for adenylate cyclase-cAMP and PKA in the extension of cilia. In synovial fibroblasts, the inhibition of adenylate cyclase, by lithium, was shown to elongate cilia [35]. Moreover, in these studies, the activity of adenylate cyclase interfered with the effects of lithium on cilia length. Conversely, however, in kidney and bone cells, the activation of adenylate cyclase by forskolin resulted in cilia elongation [14]. Our results indicate firmly that PKC, MEK–ERK, and PKA are involved in IL-1-induced length increases (Fig. 3), but the role for cAMP was not found (Fig. 4). We found that both introduction of an analogue of cAMP and inhibition of adenylate cyclase induced cilia elongation both in isolation and supplementary to the effects of IL-1. We found inhibition of upstream Gαi subunits also elongated cilia in isolation, but these effects did not supplement those of IL-1. This may indicate a shared role of G-protein subunits in IL-1 and adenylate cyclase mechanisms. However, importantly, we see no evidence to suggest that IL-1-induced elongation and cAMP-induced elongation are linked upstream of PKA (Fig. 6). The complex roles of adenylate cyclase isoforms localized to the cilium [35] and localized changes in cAMP may explain the apparent conflicts from previous studies. Adenylate cyclase isoforms are exhibited by chondrocytes [42], and we have shown that the system is active in chondrocytes where a cAMP analogue elongates the cilium in a dose-dependent manner acting through PKA, but have no evidence for a relevance of this in the context of Inflammation.

We thus hypothesize that, in response to IL-1, downstream regulation of anterograde IFT is conducted by PKA, as has been shown previously with fluid flow mediated changes in cilia length [14]. Indeed, the cilia role of PKA is well established, as PKA has been linked to cilia-dependent signaling systems including hedgehog and polycystin signaling [43–46]. In addition to the role of PKA, we have established that PKC and the MEK–ERK kinases are both involved in IL-1-stimulated elongation, perhaps a further indication of the complexity of IL-1-elicited cellular responses. Other potential candidates involved in IFT and IL-1-induced cilia elongation include hypoxia inducible factor (HIF), [28, 47, 48], cytosolic calcium, and the actin cytoskeleton [14, 22, 23], all of which are influenced by interleukins [49]. However, this is not to exclude additional reported mechanisms including FGF signaling [50], the Dcdc2 protein [13], and the tubulin cytoskeleton itself [23]. We believe that the significance of IL-1 regulation of ciliary length may lie with hedgehog signaling alterations as supported by the correlation between structural changes, including length, and hedgehog signal transduction [36].

To investigate the role of IFT and primary cilia in downstream inflammatory signaling, the present study utilized an IFT88 mutant chondrocyte model. The Tg737^*ORPK*^ model knocks out the IFT88 gene, disrupting polaris expression, and rendering cells with stunted [10] or, in the case of chondrocytes here, no obvious cilia structure (Fig. 5b). In response to IL-1, many cells including chondrocytes exhibit induction of nitric oxide synthase (iNOS) and cyclooxygenase 2 (COX-2) expression triggering the release of the potent inflammatory chemokines, nitric oxide (NO) and prostaglandin (PGE_2_). Here, we show that loss of primary cilia and IFT88 had no effect on basal NO and PGE_2_, but significantly attenuated the normal up-regulation observed in WT cells in response to IL-1β (Fig. 5). This may explain why mechanical loading, which is known to reduce cilia length in chondrocyte [30], also down-regulates the IL-1β-induced release of NO and PGE_2_ [30, 51]. It is also likely that IL-1β-induced cilia elongation will influence other aspects of cilia function including mechanotransduction [9, 14, 15] and hedgehog signaling which has already been linked to arthritis [27]. We finally examined the link between IL-1-induced cilia elongation via PKA (Fig. 3a) and the role of IFT88 (Fig. 5c, d) in the inflammatory response (Fig. 5e, f). We show that selective inhibition of PKA also inhibits PGE_2_ and NO release in bovine chondrocytes treated with IL-1. Thus, the cilium and associated elongation in response to IL-1 play critical roles in the activation of inflammatory chemokine signaling. The primary cilium already has central roles throughout cell biology, but here we propose, for the first time, that the cilium and the regulation of its structure and function are of fundamental importance in inflammation. The localized and specific nature of the cilia proteome governing maintenance and function of the primary cilia provides exciting potential therapeutic targets for inflammatory conditions.

## References

[1] Goetz SC, Anderson KV (2010). The primary cilium: a signalling centre during vertebrate development. Nat Rev Genet.

[2] Tummala P, Arnsdorf EJ, Jacobs CR (2010). The role of primary cilia in mesenchymal stem cell differentiation: a pivotal switch in guiding lineage commitment. Cell Mol Bioeng.

[3] Kim S, Zaghloul NA, Bubenshchikova E, Oh EC, Rankin S, Katsanis N, Obara T, Tsiokas L (2011). Nde1-mediated inhibition of ciliogenesis affects cell cycle re-entry. Nat Cell Biol.

[4] Michaud EJ, Yoder BK (2006). The primary cilium in cell signaling and cancer. Cancer Res.

[5] Schneider L, Cammer M, Lehman J, Nielsen SK, Guerra CF, Veland IR, Stock C, Hoffmann EK, Yoder BK, Schwab A, Satir P, Christensen ST (2010). Directional cell migration and chemotaxis in wound healing response to PDGF-AA are coordinated by the primary cilium in fibroblasts. Cell Physiol Biochem.

[6] Malone AM, Anderson CT, Tummala P, Kwon RY, Johnston TR, Stearns T, Jacobs CR (2007). Primary cilia mediate mechanosensing in bone cells by a calcium-independent mechanism. Proc Natl Acad Sci USA.

[7] Hovater MB, Olteanu D, Hanson EL, Cheng NL, Siroky B, Fintha A, Komlosi P, Liu W, Satlin LM, Bell PD, Yoder BK, Schwiebert EM (2008). Loss of apical monocilia on collecting duct principal cells impairs ATP secretion across the apical cell surface and ATP-dependent and flow-induced calcium signals. Purinergic Signal.

[8] Nauli SM, Kawanabe Y, Kaminski JJ, Pearce WJ, Ingber DE, Zhou J (2008). Endothelial cilia are fluid shear sensors that regulate calcium signaling and nitric oxide production through polycystin-1. Circulation.

[9] Wann AK, Zuo N, Haycraft CJ, Jensen CG, Poole CA, McGlashan SR, Knight MM (2012) Primary cilia mediate mechanotransduction through control of ATP-induced Ca^2+^ signaling in compressed chondrocytes. FASEB J 26:1663–1671 10.1096/fj.11-193649 PMC3316893 22223751

[10] Pazour GJ, Dickert BL, Vucica Y, Seeley ES, Rosenbaum JL, Witman GB, Cole DG (2000). Chlamydomonas IFT88 and its mouse homologue, polycystic kidney disease gene tg737, are required for assembly of cilia and flagella. J Cell Biol.

[11] Lancaster MA, Gleeson JG (2009). The primary cilium as a cellular signaling center: lessons from disease. Curr Opin Genet Dev.

[12] Mokrzan EM, Lewis JS, Mykytyn K (2007). Differences in renal tubule primary cilia length in a mouse model of Bardet–Biedl syndrome. Nephron Exp Nephrol.

[13] Massinen S, Hokkanen ME, Matsson H, Tammimies K, Tapia-Paez I, Dahlstrom-Heuser V, Kuja-Panula J, Burghoorn J, Jeppsson KE, Swoboda P, Peyrard-Janvid M, Toftgard R, Castren E, Kere J (2011). Increased expression of the dyslexia candidate gene DCDC2 affects length and signaling of primary cilia in neurons. PLoS ONE.

[14] Besschetnova TY, Kolpakova-Hart E, Guan Y, Zhou J, Olsen BR, Shah JV (2010). Identification of signaling pathways regulating primary cilium length and flow-mediated adaptation. Curr Biol.

[15] Abdul-Majeed S, Moloney BC, Nauli SM (2011). Mechanisms regulating cilia growth and cilia function in endothelial cells. Cell Mol Life Sci.

[16] Rosenbaum JL, Moulder JE, Ringo DL (1969). Flagellar elongation and shortening in Chlamydomonas. The use of cycloheximide and colchicine to study the synthesis and assembly of flagellar proteins. J Cell Biol.

[17] Asleson CM, Lefebvre PA (1998). Genetic analysis of flagellar length control in *Chlamydomonas reinhardtii*: a new long-flagella locus and extragenic suppressor mutations. Genetics.

[18] Engel BD, Ludington WB, Marshall WF (2009). Intraflagellar transport particle size scales inversely with flagellar length: revisiting the balance-point length control model. J Cell Biol.

[19] Mukhopadhyay S, Lu Y, Shaham S, Sengupta P (2008). Sensory signaling-dependent remodeling of olfactory cilia architecture in *C. elegans*. Dev Cell.

[20] Lefebvre PA, Nordstrom SA, Moulder JE, Rosenbaum JL (1978). Flagellar elongation and shortening in Chlamydomonas. IV. Effects of flagellar detachment, regeneration, and resorption on the induction of flagellar protein synthesis. J Cell Biol.

[21] Cao M, Li G, Pan J (2009). Regulation of cilia assembly, disassembly, and length by protein phosphorylation. Methods Cell Biol.

[22] Kim J, Lee JE, Heynen-Genel S, Suyama E, Ono K, Lee K, Ideker T, Aza-Blanc P, Gleeson JG (2010). Functional genomic screen for modulators of ciliogenesis and cilium length. Nature.

[23] Sharma N, Kosan ZA, Stallworth JE, Berbari NF, Yoder BK (2011). Soluble levels of cytosolic tubulin regulate ciliary length control. Mol Biol Cell.

[24] Deane JA, Ricardo SD (2012). Emerging roles for renal primary cilia in epithelial repair. Int Rev Cell Mol Biol.

[25] Jensen CG, Poole CA, McGlashan SR, Marko M, Issa ZI, Vujcich KV, Bowser SS (2004). Ultrastructural, tomographic and confocal imaging of the chondrocyte primary cilium in situ. Cell Biol Int.

[26] McGlashan SR, Cluett EC, Jensen CG, Poole CA (2008). Primary cilia in osteoarthritic chondrocytes: from chondrons to clusters. Dev Dyn.

[27] Lin AC, Seeto BL, Bartoszko JM, Khoury MA, Whetstone H, Ho L, Hsu C, Ali SA, Alman BA (2009). Modulating hedgehog signaling can attenuate the severity of osteoarthritis. Nat Med.

[28] Verghese E, Zhuang J, Saiti D, Ricardo SD, Deane JA (2011). In vitro investigation of renal epithelial injury suggests that primary cilium length is regulated by hypoxia-inducible mechanisms. Cell Biol Int.

[29] Dinarello CA (2011). A clinical perspective of IL-1beta as the gatekeeper of inflammation. Eur J Immunol.

[30] Chowdhury TT, Bader DL, Lee DA (2001). Dynamic compression inhibits the synthesis of nitric oxide and PGE(2) by IL-1beta-stimulated chondrocytes cultured in agarose constructs. Biochem Biophys Res Commun.

[31] Yoder BK, Richards WG, Sommardahl C, Sweeney WE, Michaud EJ, Wilkinson JE, Avner ED, Woychik RP (1997). Differential rescue of the renal and hepatic disease in an autosomal recessive polycystic kidney disease mouse mutant. A new model to study the liver lesion. Am J Pathol.

[32] McGlashan SR, Jensen CG, Poole CA (2006). Localization of extracellular matrix receptors on the chondrocyte primary cilium. J Histochem Cytochem.

[33] Lee DA, Frean SP, Lees P, Bader DL (1998). Dynamic mechanical compression influences nitric oxide production by articular chondrocytes seeded in agarose. Biochem Biophys Res Commun.

[34] Kwon RY, Temiyasathit S, Tummala P, Quah CC, Jacobs CR (2010). Primary cilium-dependent mechanosensing is mediated by adenylyl cyclase 6 and cyclic AMP in bone cells. FASEB J.

[35] Ou Y, Ruan Y, Cheng M, Moser JJ, Rattner JB, van der Hoorn FA (2009). Adenylate cyclase regulates elongation of mammalian primary cilia. Exp Cell Res.

[36] Tran PV, Haycraft CJ, Besschetnova TY, Turbe-Doan A, Stottmann RW, Herron BJ, Chesebro AL, Qiu H, Scherz PJ, Shah JV, Yoder BK, Beier DR (2008). THM1 negatively modulates mouse sonic hedgehog signal transduction and affects retrograde intraflagellar transport in cilia. Nat Genet.

[37] Verghese E, Ricardo SD, Weidenfeld R, Zhuang J, Hill PA, Langham RG, Deane JA (2009). Renal primary cilia lengthen after acute tubular necrosis. J Am Soc Nephrol.

[38] McGlashan SR, Knight MM, Chowdhury TT, Joshi P, Jensen CG, Kennedy S, Poole CA (2010). Mechanical loading modulates chondrocyte primary cilia incidence and length. Cell Biol Int.

[39] Gardner K, Arnoczky SP, Lavagnino M (2011). Effect of in vitro stress-deprivation and cyclic loading on the length of tendon cell cilia in situ. J Orthop Res.

[40] Palmer KJ, MacCarthy-Morrogh L, Smyllie N, Stephens DJ (2011). A role for Tctex-1 (DYNLT1) in controlling primary cilium length. Eur J Cell Biol.

[41] Espanol AJ, Goren N, Ribeiro ML, Sales ME (2010). Nitric oxide synthase 1 and cyclooxygenase-2 enzymes are targets of muscarinic activation in normal and inflamed NIH3T3 cells. Inflamm Res.

[42] Memon I, Khan KM, Siddiqui S, Perveen S, Ishaq M (2010). Temporal expression of calcium/calmodulin-dependent adenylyl cyclase isoforms in rat articular chondrocytes: RT-PCR and immunohistochemical localization. J Anat.

[43] Tukachinsky H, Lopez LV, Salic A (2010). A mechanism for vertebrate Hedgehog signaling: recruitment to cilia and dissociation of SuFu-Gli protein complexes. J Cell Biol.

[44] Chen Y, Yue S, Xie L, Pu XH, Jin T, Cheng SY (2011). Dual Phosphorylation of suppressor of fused (Sufu) by PKA and GSK3beta regulates its stability and localization in the primary cilium. J Biol Chem.

[45] Choi YH, Suzuki A, Hajarnis S, Ma Z, Chapin HC, Caplan MJ, Pontoglio M, Somlo S, Igarashi P (2011). Polycystin-2 and phosphodiesterase 4C are components of a ciliary A-kinase anchoring protein complex that is disrupted in cystic kidney diseases. Proc Natl Acad Sci USA.

[46] Barzi M, Berenguer J, Menendez A, Alvarez-Rodriguez R, Pons S (2010). Sonic-hedgehog-mediated proliferation requires the localization of PKA to the cilium base. J Cell Sci.

[47] Toffoli S, Feron O, Raes M, Michiels C (2007). Intermittent hypoxia changes HIF-1alpha phosphorylation pattern in endothelial cells: unravelling of a new PKA-dependent regulation of HIF-1alpha. Biochim Biophys Acta.

[48] Qian D, Lin HY, Wang HM, Zhang X, Liu DL, Li QL, Zhu C (2004). Normoxic induction of the hypoxic-inducible factor-1 alpha by interleukin-1 beta involves the extracellular signal-regulated kinase 1/2 pathway in normal human cytotrophoblast cells. Biol Reprod.

[49] Pritchard S, Guilak F (2006). Effects of interleukin-1 on calcium signaling and the increase of filamentous actin in isolated and in situ articular chondrocytes. Arthritis Rheum.

[50] Neugebauer JM, Amack JD, Peterson AG, Bisgrove BW, Yost HJ (2009). FGF signalling during embryo development regulates cilia length in diverse epithelia. Nature.

[51] Agarwal S, Long P, Gassner R, Piesco NP, Buckley MJ (2001). Cyclic tensile strain suppresses catabolic effects of interleukin-1beta in fibrochondrocytes from the temporomandibular joint. Arthritis Rheum.

